# A meta-analysis comparing short-term weight and cardiometabolic changes between olanzapine/samidorphan and olanzapine

**DOI:** 10.1038/s41598-021-87285-w

**Published:** 2021-04-07

**Authors:** Manit Srisurapanont, Sirijit Suttajit, Surinporn Likhitsathian, Benchalak Maneeton, Narong Maneeton

**Affiliations:** Department of Psychiatry, Faculty of Medicine, Chiang Mai University, 110 Inthawarorot Road, Si Phum, Mueang, Chiang Mai, 50200 Thailand

**Keywords:** Schizophrenia, Drug safety, Pharmacology

## Abstract

This study compared weight and cardiometabolic changes after short-term treatment of olanzapine/samidorphan and olanzapine. Eligible criteria for an included trial were ≤ 24 weeks, randomized controlled trials (RCTs) that compared olanzapine/samidorphan and olanzapine treatments in patients/healthy volunteers and reported weight or cardiometabolic outcomes. Three databases were searched on October 31, 2020. Primary outcomes included weight changes and all-cause dropout rates. Standardized mean differences (SMDs) and risk ratios (RRs) were computed and pooled using a random-effect model. This meta-analysis included four RCTs (n = 1195). The heterogeneous data revealed that weight changes were not significantly different between olanzapine/samidorphan and olanzapine groups (4 RCTs, SDM = − 0.19, 95% CI − 0.45 to 0.07, *I*^2^ = 75%). The whole-sample, pooled RR of all-cause dropout rates (4 RCTs, RR = 1.02, 95% CI 0.84 to 1.23, *I*^2^ = 0%) was not significant different between olanzapine/samidorphan and olanzapine groups. A lower percentage of males and a lower initial body mass index were associated with the greater effect of samidorphan in preventing olanzapine-induced weight gain. Current evidence is insufficient to support the use of samidorphan to prevent olanzapine-induced weight gain and olanzapine-induced cardiometabolic abnormalities. Samidorphan is well accepted by olanzapine-treated patients.

## Introduction

Olanzapine is one of the most studied and widely used second-generation antipsychotics (SGAs). For schizophrenia, it is effective and has a longer time to all-cause discontinuation than many antipsychotic medications^1,2^. Olanzapine can effectively control acute mania and reduce the overall risk of relapses^3,4^. Its combination with fluoxetine also performs well in managing bipolar depression^5^. These findings suggest the favorable efficacy of olanzapine for the treatment of schizophrenia and bipolar disorder.

Weight gain and metabolic syndrome are serious adverse effects associated with olanzapine treatment. Olanzapine is one of the common medications with a high propensity to induce weight gain and metabolic syndrome^6,7^. In patients with schizophrenia, it may cause weight gain for a mean of 3.3 kg within eight weeks of treatment^8^. Except for clozapine, olanzapine is associated with more severe metabolic adverse events than other SGAs^9^. Metformin, topiramate, and aripiprazole may modestly reduce weight gain in olanzapine-treated patients^10,11^. The adverse consequences of weight gain and metabolic syndrome are major concerns of olanzapine treatment.

The opioid system affects eating behavior and body weight regulation. While opioid agonists increase food intake and body weight gain, opioid antagonists can induce weight loss^12^. Of many opioid antagonists, samidorphan (3-carboxamido-4-hydroxynaltrexone) is structurally related to the mixed-opioid-receptor antagonist naltrexone but has a five-fold greater affinity at mu-opioid receptors^13^. It also has much greater bioavailability when administered orally. Samidorphan is, therefore, a potential medication for treating obesity and preventing weight gain.

Several lines of evidence suggest that samidorphan has no deleterious effect on olanzapine-treated patients. First, samidorphan has no drug-drug interaction with olanzapine^14^. Second, opioid antagonists are safe for schizophrenia and may modestly reduce psychotic symptoms^15^. Third, olanzapine/samidorphan is superior to placebo and as effective as olanzapine for the treatment of schizophrenia^16^.

There has been a development of olanzapine/samidorphan combination treatment for patients appropriate for olanzapine treatment. Therefore, we conducted this systematic review of randomized controlled trials (RCTs) to compare the short-term efficacy, tolerability, and acceptability between olanzapine/samidorphan and olanzapine. The efficacy being focused in this review includes weight gain and cardiometabolic changes.

## Methods

### Protocol and registration

The protocol of this systematic review was prospectively registered at Open Science Framework (OSF) (https://osf.io/g49sk/) under the title of “Systematic Review of Samidorphan for Olanzapine-Induced Weight Gain and Metabolic Disturbances: a Pre-Registration Protocol”. This review was prepared in accordance with the PRISMA Statement for Reporting Systematic Reviews and Meta-Analyses of Studies That Evaluate Health Care Interventions (see Appendix 1 in Supplementary Information)^17^.

### Eligibility criteria, information sources, and searches

The criteria for an included trial were as follows: (i) Participants—patients or healthy volunteers; (ii) Intervention—olanzapine/samidorphan combination; (iii) Comparator—olanzapine/placebo or olanzapine monotherapy; (iv) Outcomes of interest—weight, fasting plasma glucose (FPG), high-density cholesterol (HDL), low-density cholesterol (LDL), and triglyceride (TG); (v) Study designs—randomized controlled trials in in- or out-patients; and (vi) Study duration—24 weeks or less.

Database searches included Pubmed, Embase, and Cochrane Central Register of Controlled Trials. The search duration covered from the inception of each database to October 31, 2020. We searched Clinicaltrial.gov and EudraCT to screen for relevant rials. Appendix 2 in Supplementary Information shows the search details. No limitation of publication years, languages, or publication status was applied.

### Study selection, data, and risks of bias in individual trials

MS and SS independently screened the titles and abstracts, evaluated the full-text publications, selected the trials, and assessed the trial quality. If there was any discrepancy, these two reviewers resolved it using a consensus discussion.

We developed a data extraction form. MS extracted the trial information and data as follows: (i) study details, including publication year, study duration, placebo used; (ii) characteristics of trial participants, including age, % male, diagnosis, initial weight/body mass index (BMI); (iii) intervention details, including olanzapine and samidorphan doses; (iv) outcome details, including available outcomes and their results. SS rechecked the extracted data.

The primary outcomes of efficacy and acceptability were weight changes and all-cause dropout rates, respectively. Secondary efficacy outcomes were the changes of FPG, HDL, LDL, and TG. Tolerability was assessed using adverse dropout rates (the dropout rates due to adverse events). For the outcome being assessed at several time points, we extracted only the final outcomes. All outcomes reported after treatment discontinuation, the end of RCT, or 24 weeks of treatment were disregards.

We assessed the quality of included trials using the Cochrane risk-of-bias tool for randomized trials, version 2 (RoB2)^18^. Five domains of bias being assessed included randomization processes, adherence to the assigned interventions, missing outcome data, the bias of measurement, and the bias of reported results. Each domain was rated as low risk-of-bias, some concerns, or high risk-of-bias. The worst risk-of-bias in any of these domains was used to rate the overall risk of bias.

### Summary of measures and analysis methods

We compared the outcomes between olanzapine/samidorphan and olanzapine groups. The continuous and dichotomous data were compared using the standardized mean differences (SMDs) and the risk ratios (RRs), respectively. We pooled the SMDs using Hedges’ g statistics^19^. Both SMDs and RRs were aggregated using the inverse-variance, random-effects meta-analyses based on the DerSimonian and Laird methods^20^. The SMD of 0.2, 0.5, or 0.8 was considered as a small, medium, or large effect size, repsectively^21^. The heterogeneity of each dataset was visualized using the forest plot and quantified using the *I*^2^ statistics^22^. An *I*^2^ value of 75% or higher was rated as considerable heterogeneity.

### Risks of bias across trials and additional analyses

We conducted subgroup analyses based on the participants’ characteristics, e.g., diagnosis. The publication bias of each outcome was visualized using the funnel plot. We quantified the publication bias if the number of trials was larger than 10^22^.

For the outcome with considerable heterogeneity, we conducted meta-regression analyses to explore the moderating effects of study duration, initial BMI, mean age, and the percentage of male participants.

### Assessing the quality of cumulative evidence

The quality of cumulative evidence derived from current meta-analyses was assessed using the GRADE approach^23^. This quality reflected the confidence in the treatment-effect estimate (pooled SMD and RR) and was rated as high, moderate, low, or very low level. The estimate drawn from a RCT dataset was initially rated as high-quality evidence but would be downgraded if the dataset had the following concerns: (i) high risk of bias, (ii) high inconsistency (considerable heterogeneity), (iii) indirectness (not obtained from a direct group), (iv) imprecision (a non-significant result), and (v) high or indeterminable publication bias. In this study, the presence of each concern would lead to the downgrading of evidence quality by one level.

### Statistical software

We analyzed and visualized the data using the *Meta* package version 4.15 under the *R* Program version 4.0 and the *Rstudio* software version 1.3^24–26^.

## Results

### Study selection, study characteristics, and risk of bias within trials

**Figure 1:**
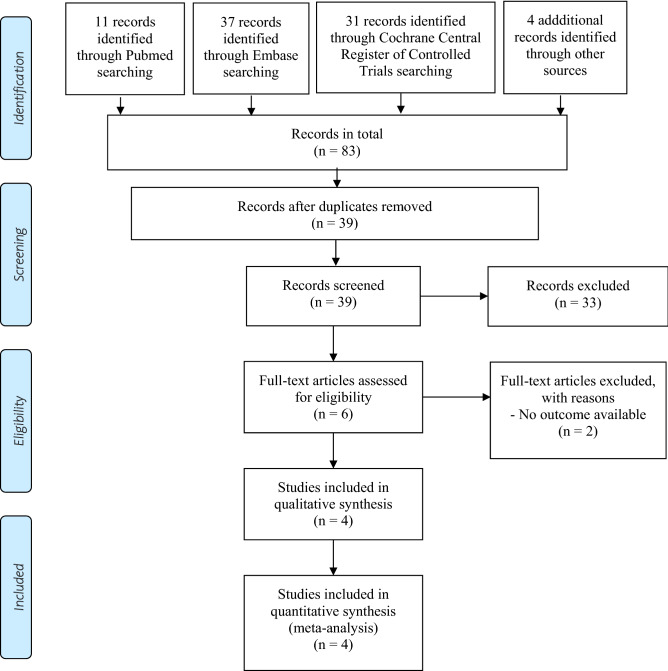
PRISMA flow diagram: records retrieved from database searches and trial inclusion in the systematic review of randomized controlled trials comparing olanzapine/samidorphan and olanzapine for mitigating olanzapine-induced weight gain and cardiometabolic disturbances.

The searches at Pubmed, Embase, Cochrane Central Register of Controlled Trials, and additional sources retrieved a total of 83 records (see Fig. 1). After the duplication removal and title/abstract screening, we examined six full-text articles. Two articles were excluded because the outcomes of interest were not reported^27,28^.

**Figure 2:**
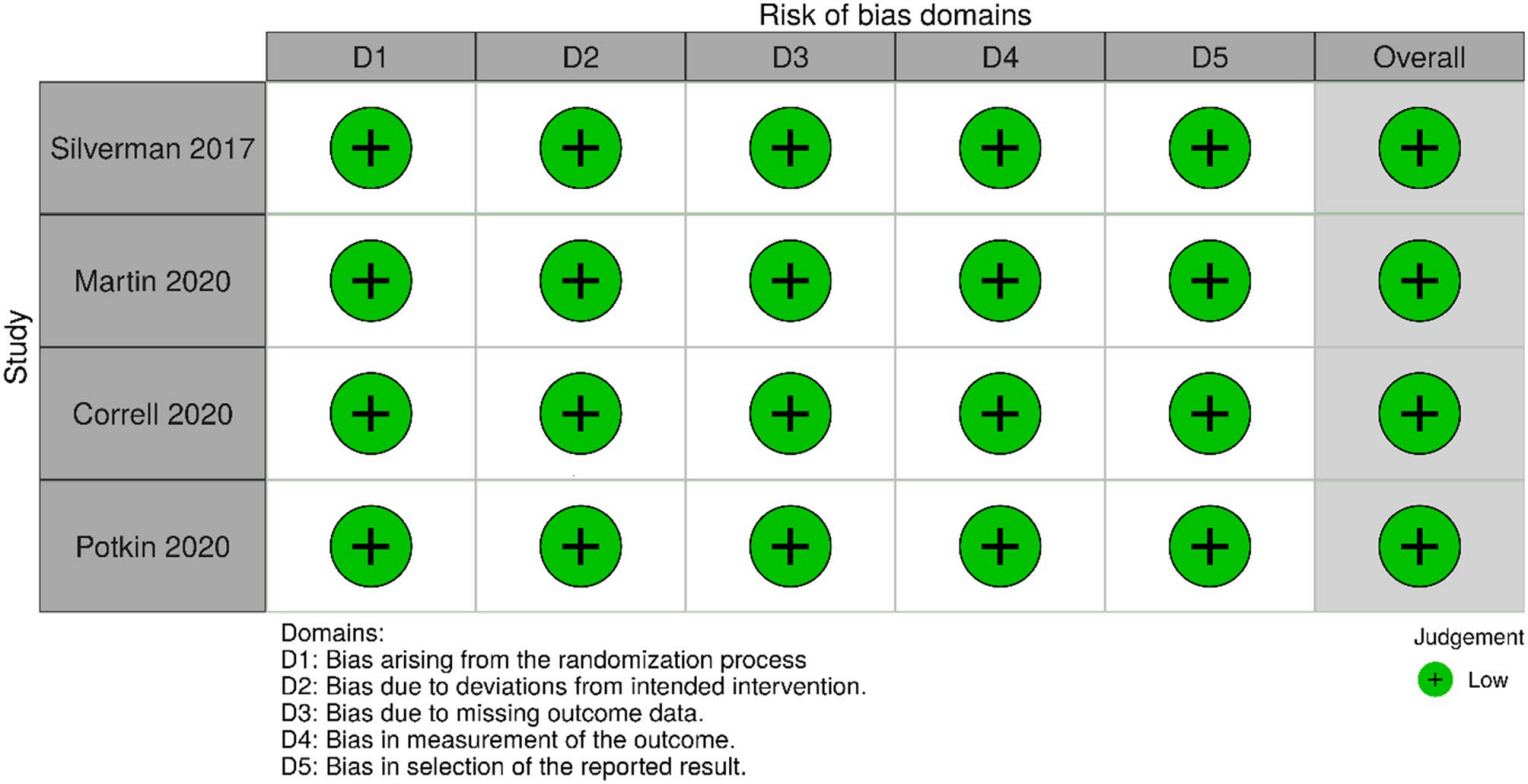
Risk of bias within four trials included in the meta-analysis of randomized controlled trials comparing olanzapine/samidorphan and olanzapine for mitigating olanzapine-induced weight gain and cardiometabolic disturbances.

This systematic review included one trial in healthy volunteers and three trials in patients with schizophrenia (n = 1195)^16,29–31^ (see Table 1). All were industry-sponsored, double-blind, randomized controlled trials with a study duration of 3–24 weeks. Most participants were male (62%-100%) and aged between 18 and 70 years (mean age of 26.4–41.2 years). Means of the initial BMI and weight were 22.6–26.9 kg/m^2^ and 68–80 kg, respectively. Both olanzapine and samidorphan were given at the doses of 5–20 mg/day. While three trials reported all seven outcomes^29–31^, one trial provided only three outcomes of interest (i.e., weight change, all-cause dropout rates, and adverse dropout rates)^16^. All four trials had low risks of bias across the five assessment domains (see Fig. 2). All included trials were, therefore, rated as low risks of overall bias.

**Table 1:** Characteristics of randomized controlled trials comparing weight and cardiometabolic changes after short-term treatment of olanzapine/SAM and olanzapine [arranged by year of publication].

Author (year), country	Study design (Sponsor)	Participant	Intervention (mg/day)	Comparator (mg/day)	Outcome and result ( OLZ/SAM vs. OLZ)
Silverman (2018), USA	DBRT, placebo-controlled, 3 weeks (Alkermes)	Aged 18–40 years (average = 26.44 years) Male: 100% Diagnosis: Healthy BMI: 18–25 kg/m^2^ Mean initial BMI and weight: 22.55 kg/m^2^ and 67.80 kg	OLZ/SAM = 10/5 mg/day (n = 34)	OLZ = 10 mg/day (n = 35)	Weight change (mean kg): 2.2 vs. 3.1 (p = 0.02) FPG change (mean mmol/L): − 0.1 vs. 0.0 (p = 0.70) HDL change (mean mmol/L): − 0.1 vs. 0.0 (p = 0.41) LDL change (mean mmol/L): 0.1 vs. 0.2 (p = 0.50) TG change (mean mmol/L): 0.2 vs. 0.4 (p = 0.28) All-cause dropouts (n): 7 vs. 6 (p, N/A) Adverse dropouts (n): 3 vs. 3 (p, N/A)
Martin (2019), Multi-country	DBRT, placebo-controlled, 12 weeks (Alkermes)	Aged 18–50 years (average = 38.86 years) Male: 73.8% Diagnosis: Schizophrenia Mean PANSS total score: 62 BMI: 17–30 kg/m^2^ Mean initial BMI and weight: 25.18 kg/m^2^ and 76.91 kg	OLZ/SAM = 5–20/5–20 mg/day (n = 235)	OLZ = 5–20 mg/day (n = 75)	Weight change (mean kg): 1.9 vs. 2.2 (p = 0.02) FPG change (mean mg/dL): 5.4 vs. 4.1 (p, N/A) HDL change (mean mg/dL): − 2.1 vs. − 3.2 (p, N/A) LDL change (mean mg/dL): 7.4 vs. 11.5 (p, N/A) TG change (mean mg/dL): 4.0 vs. 6.5 (p, N/A) All-cause dropouts (n): 69 vs. 19 (p, N/A) Adverse dropouts (n): 21 vs. 3 (p, N/A)
Correll (2020), USA	DBRT, 24 weeks (Alkermes)	Aged 18–55 years (average = 40.20 years) Male: 72.7% Diagnosis: Schizophrenia Mean PANSS total score: 69.2 BMI: 18–30 kg/m^2^ Mean initial BMI and weight: 25.45 kg/m^2^ and 77.37 kg	OLZ/SAM = 10–20/10 mg/day (n = 274)	OLZ = 10–20 mg/day (n = 276)	Weight change (LSM %): 4.21 vs. 6.59 (p = 0.003) FPG change (mean mg/dL): 4.5 vs. 2.3 (p, N/A) HDL change (mean mg/dL): − 5.1 vs. − 4.5 (p, N/A) LDL change (mean mg/dL): 0.6 vs. 0.9 (p, N/A) TG change (mean mg/dL): 23.9 vs. 24.5 (p, N/A) All-cause dropouts (n): 98 vs. 100 (p, N/A) Adverse dropouts (n): 33 vs. 27 (p, N/A)
Potkin (2020), USA and Europe	DBRT, placebo-controlled, 4 weeks (Alkermes)	Aged 18–70 years (average = 41.15 years) Male: 62.2% Diagnosis: Schizophrenia Mean PANSS total score: 101.2 BMI: 18–40 kg/m^2^ Mean initial BMI and weight: 26.90 kg/m^2^ and 80.04 kg	OLZ/SAM = 10–20/10 mg/day (n = 134)	OLZ = 10–20 mg/day (n = 133)	Weight change (mean kg): 3.02 vs. 2.38 (p, N/A) All-cause dropouts (n): 12 vs. 14 (p, N/A) Adverse dropouts (n): 2 vs. 2 (p, N/A)

*DBRT* Double-blind, randomized trial, *PANSS* Positive and Negative Syndrome Scale, *OLZ* Olanzapine, *SAM* Samidorphan, *FPG* Fasting plasma glucose, *HDL* High-density cholesterol, *LDL* Low-density cholesterol, *TG* Triglyceride, *BMI* body mass index, *N/A* not available.

### Results of individual trials

Three of four included trials found that olanzapine/samidorphan was significantly superior to olanzapine in mitigating weight gain (see Table 1)^29–31^. The last trial found that participants taking olanzapine/samidorphan gained more weight than olanzapine-treated participants (3.02 kg vs. 2.38 kg)^16^. No trial found any significant difference between treatment groups regarding the changes of FPG, HDL, LDL, and TG, as well as adverse and all-cause dropout rates.

### Synthesis of results

We compared the differences between treatment groups within the whole sample, the healthy-volunteer subgroup, and the subgroup of patients with schizophrenia for all outcomes.

#### Weight changes

**Figure 3:**
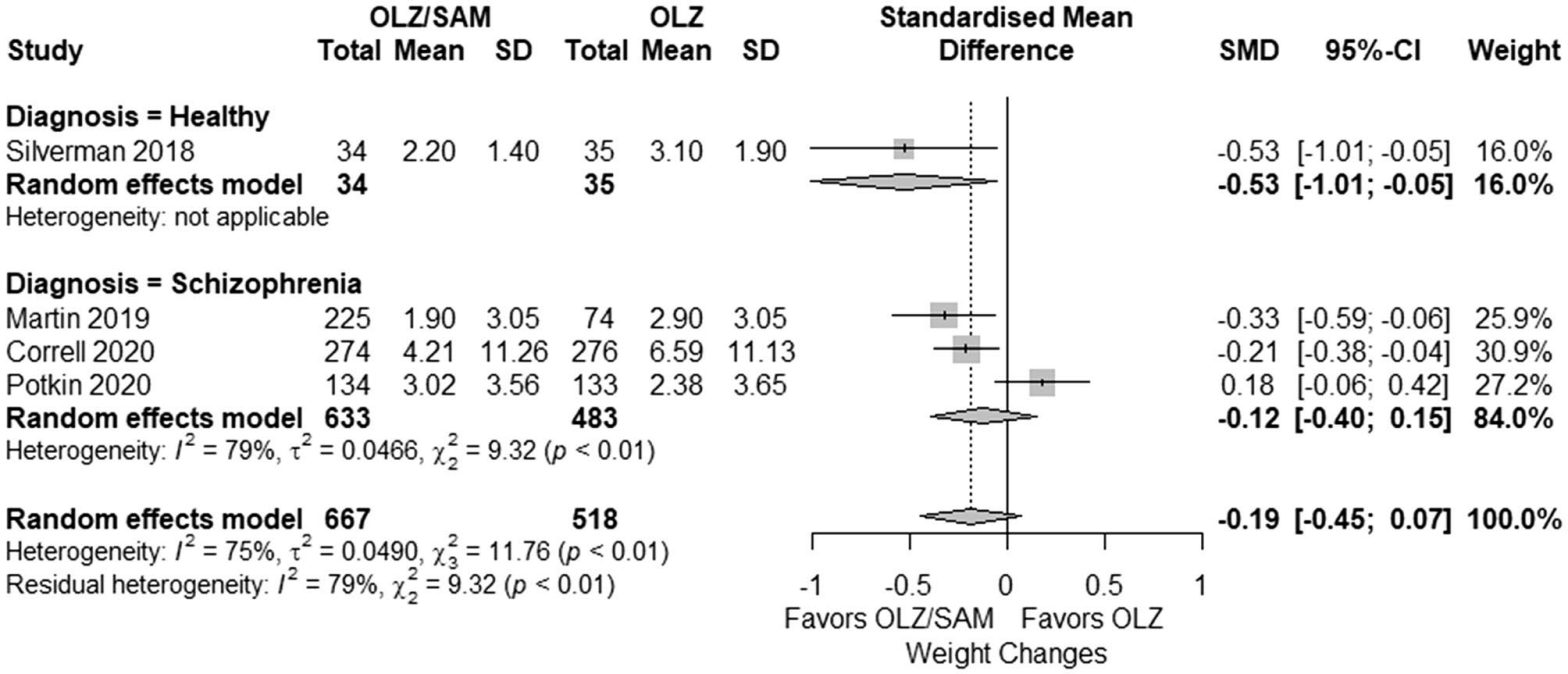
Subgroup meta-analysis (healthy individuals vs. patients with schizophrenia) of weight changes expressed as the standardized mean differences comparing between olanzapine/samidorphan and olanzapine. The upper two diamonds indicate the pooled results of healthy and schizophrenia subgroups, respectively. The diamond at the bottom shows the pooled results of all trials.

The heterogeneous data revealed that the whole-sample, pooled SMD of weight change was not significantly different between olanzapine/samidorphan and olanzapine groups (4 RCTs, SDM = − 0.19, 95% CI − 0.45 to 0.07, *I*^2^ = 75%) (see Fig. 3). The effect of samidorphan in preventing weight gain was significantly greater in the healthy subgroup compared to the schizophrenia subgroup (Q χ^2^ = 9.32, p < 0.01). In the healthy subgroup, olanzapine/samidorphan was associated with significantly less weight gain than olanzapine (1 RCT, SMD = − 0.53, 95% CI − 1.01 to − 0.05). In the schizophrenia subgroup, the heterogeneous data revealed that the pooled weight change of three included trials was not significantly different between groups (3 RCTs, SMD = − 0.12, 95% CI = − 0.40 to 0.15, *I*^2^ = 79%).

#### Cardiometabolic outcomes

Appendices 3–6 show the forest plots of all four cardiometabolic outcomes. The whole-sample, pooled SMDs were not significantly different between olanzapine/samidorphan and olanzapine groups (see Table 2). Those included the changes of FPG (3 RCTs, SMD = 0.09, 95% CI − 0.04 to 0.23, *I*^2^ = 0%), HDL (3 RCTs, SMD = − 0.07, 95% CI − 0.32 to 0.17, *I*^2^ = 57%), LDL (3 RCTs, SMD = − 0.08, 95% CI − 0.22 to 0.06, *I*^2^ = 1%), and TG (3 RCTs, SMD = − 0.03, 95% CI − 0.17 to 0.10, *I*^2^ = 0%). Subgroup analyses of all four cardiometabolic outcomes found no significant difference between subgroups.

**Table 2:** Summary of cardiometabolic outcomes and dropout rates.

Outcome	Subgroup and group	Number of included trials (n: OLZ/SAM vs. OLZ)	SMD/RR [95% CI]	I^2^
FPG changes	Healthy	1 (n: 34 vs. 35)	SMD = − 0.19 [− 0.67; 0.28]	N/A
	Schizophrenia	2 (n: 499 vs. 350)	SMD = 0.12 [− 0.02; 0.26]	0%
	All	3 (n: 533 vs. 385)	SMD = 0.09 [− 0.04; 0.23]	0%
HDL changes	Healthy	1 (n: 34 vs. 35)	SMD = − 0.49 [− 0.97; − 0.01]	N/A
	Schizophrenia	2 (n: 499 vs. 350)	SMD = 0.00 [− 0.14; 0.14]	0%
	All	3 (n: 533 vs. 385)	SMD = − 0.07 [− 0.32; 0.17]	0%
LDL changes	Healthy	1 (n: 34 vs. 35)	SMD = − 0.28 [− 0.75; 0.19]	N/A
	Schizophrenia	2 (n: 499 vs. 350)	SMD = − 0.07 [− 0.24; 0.09]	21%
	All	3 (n: 533 vs. 385)	SMD = − 0.08 [− 0.22; 0.06]	1%
TG changes	Healthy	1 (n: 34 vs. 35)	SMD = − 0.26 [− 0.73; 0.22]	N/A
	Schizophrenia	2 (n: 499 vs. 350)	SMD = − 0.01 [− 0.16; 0.13]	0%
	All	3 (n: 533 vs. 385)	SMD = − 0.03 [− 0.17; 0.10]	0%
All-cause dropout rates	Healthy	1 (n: 34 vs. 35)	RR = 1.20 [0.45; 3.21]	N/A
	Schizophrenia	3 (n: 642 vs. 484)	RR = 1.01 [0.83; 1.22]	0%
	All	4 (n:676 vs. 519)	RR = 1.02 [0.84; 1.23]	0%
Adverse dropout rates	Healthy	1 (n: 34 vs. 35)	RR = 1.03 [0.22; 4.75]	N/A
	Schizophrenia	3 (n: 642 vs. 484)	RR = 1.32 [0.86; 2.04]	0%
	All	4 (n:676 vs. 519)	RR = 1.30 [0.85; 1.97]	0%

*OLZ* Olanzapine, *SAM* Samidorphan, FPG Fasting plasma glucose, *HDL* High-density cholesterol, *LDL* Low-density cholesterol, *TG* Triglyceride, *BMI* body mass index, *SMD* Standardized mean difference, *RR* Risk ratio, *N/A* not available.

#### Dropout rates

Appendices 7 and 8 show the forest plots of adverse and all-cause dropout rates. The whole-sample, pooled RRs of adverse (4 RCTs, RR = 1.30, 95% CI 0.85 to 1.97, *I*^2^ = 0%) and all-cause (4 RCTs, RR = 1.02, 95% CI 0.84 to 1.23, *I*^2^ = 0%) were not significant different between olanzapine/samidorphan and olanzapine groups. Subgroup analyses of both dropout rates found no significant difference between subgroups.

### Risk of bias across trials and additional analyses

**Figure 4:**
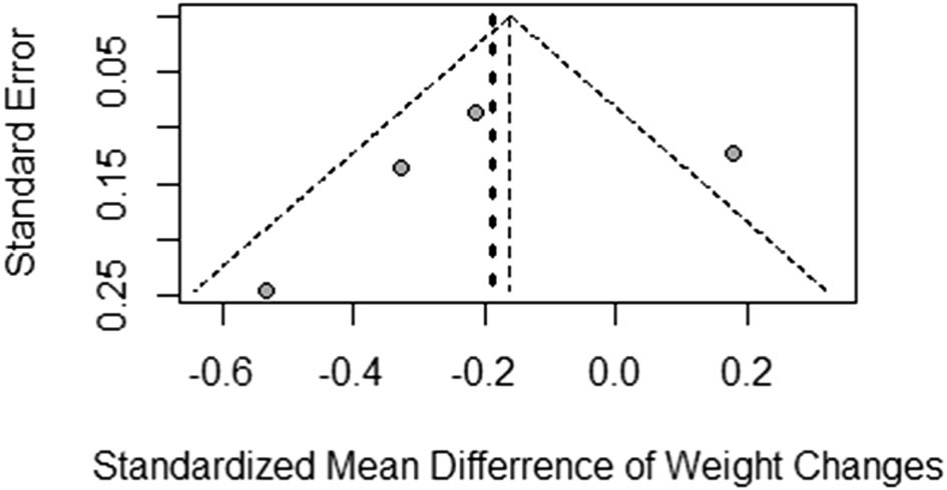
Funnel plot for assessing the publication bias of randomized controlled trials comparing olanzapine/samidorphan and olanzapine for mitigating olanzapine-induced weight gain. The bold dash line indicates the random effects estimate.

Figure 4 shows the funnel plots that explored the publication bias of weight-change trials. Appendix 9A–G shows the funnel plots exploring the publication bias of trials reporting cardiometabolic outcomes and dropout rates. No apparent asymmetry could be observed in any funnel plot. Due to the small number of trials (less than ten), we did not apply any statistical test to quantify the publication bias.

**Figure 5:**
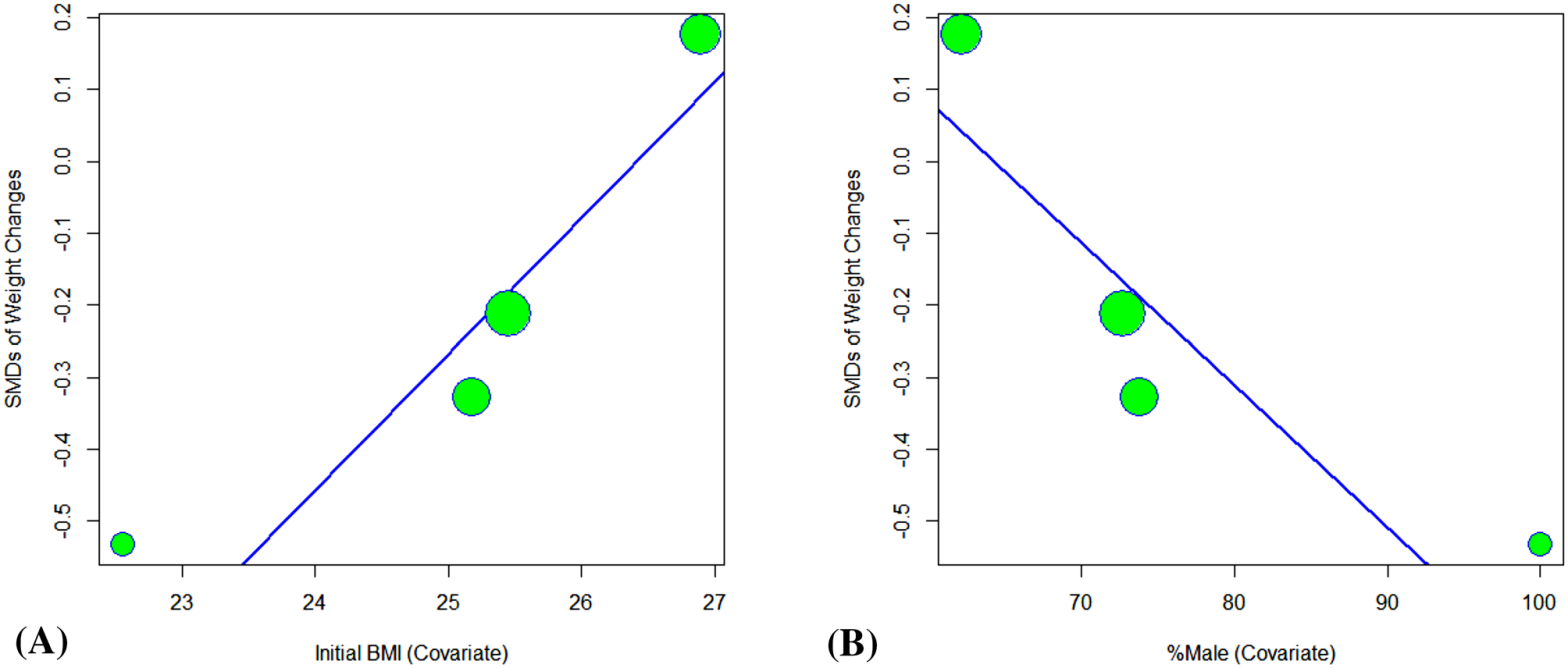
Bubble plots with fitted meta-regression lines of the SMDs of weight changes and the initial BMI (β = 0.19; p = 0.002) (**A**) or percentage of male participants (β = − 0.02; p = 0.012) (**B**). Circles are sized according to the inverses of the effect-size variances.

Due to the considerable heterogeneity of weight change data, we conducted four meta-regression analyses to explore if the weight-change effect was associated with study duration, initial BMI, mean age, or the percentage of male participants. and found that the greater effect of samidorphan in preventing olanzapine-induced weight gain was significantly associated with lower initial BMI (*β* = 0.19; p = 0.002) and lower percentages of male participants (β = − 0.02, p = 0.012) (see Fig. 5A,B). This effect of samidorphan was not significantly correlated with the study duration (β = − 0.004, p = 0.840), and the mean age of participants (β = 0.04, p = 0.138).

### Quality of cumulative evidence

Appendix 10 shows the GRADE approach used for rating the quality of cumulative evidence. This meta-analysis collected high-quality data (evidence) from RCTs. A high risk of bias was not found in any dataset (outcome). Because only 69 of 1195 participants were healthy volunteers, these minorities should not indicate the indirectness of evidence. Therefore, no indirectness concern was found in any dataset (outcome). The non-significant results (imprecision) of all datasets (outcomes) resulted in the quality downgrading of all pooled SMDs and RRs by one level. The indeterminable publication bias of all datasets (outcomes) further downgraded the quality of all pooled SMDs and RRs by one level. Considerable heterogeneity was found in the weight-change dataset and lead to the downgrading of pooled SMDs of weight change by one level. Taken together, the quality (confidence) of the pooled SMD for weight changes was downgraded by three levels from a high to very low level. For the pooled SMDs or RRs of the other outcomes, the quality of each estimate was rated down by two levels from a high to low level.

## Discussion

The effect of samidorphan in preventing olanzapine-induced weight gain is small. Current evidence is of low and very low quality and insufficient to support the use of samidorphan to prevent olanzapine-induced weight gain and olanzapine-induced cardiometabolic abnormalities. This treatment effect seems to be greater in women and individuals with lower initial BMI. The comparable adverse and all-cause dropout rates suggest that samidorphan is well tolerated and well accepted by olanzapine-treated patients.

Our meta-analysis included the weight result of a 4-week trial that was in contrast with those of the other three trials lasting 3 to 24 weeks. Potkin and colleagues (2020) found that the olanzapine/samidorphan group gained weight more than the olanzapine group for a mean difference of 0.64 kg^16^. This result was contrary to those of the other three trials that found significantly less weight gain in the olanzapine/samidorphan group^29–31^. While Potkin’s trial primarily aimed to investigate the treatment effects of olanzapine/samidorphan on psychopathology (i.e., PANSS total and subscale scores), the other three trials were designed to determine the weight-control benefit of olanzapine/samidorphan. The exclusion of Potkin’s negative results would change the non-significant benefit of olanzapine/samidorphan to a significant one.

There is a challenge in explaining the nonsignificantly more weight gain of the olazapine/samidorphan group found in Potkin’s 4-week trial. Similar to the other two trials in patients with schizophrenia^29,30^, Silverman and colleagues (2018) found that the olanzapine/samidorphan group gained weight significantly less than the olanzapine group at a mean of 0.50 kg^31^. While Silverman’s and Potkin’s trials were similar in having a short study duration (3–4 weeks), their participants were healthy individuals and patients with schizophrenia, respectively. Taken together, patients with schizophrenia might respond to the weight-gain prevention benefit of samidorphan at a slower pace than healthy individuals. The rationales behind this slow response remain unknown.

The inconsistent results of weight-gain prevention benefit of samidorphan treatment were similar to those of weight-loss benefit of naltreaxone treatment. The weight-loss benefit of naltrexone has been investigated in two trials of patients with schizophrenia and schizoaffective disorder who were on stable doses of antipsychotic medications. While one trial found this benefit in patients receiving various antipsychotic medications^32^, the other trial did not find that benefit in patients taking olanzapine^33^.

Similar to other weight-loss medications, samidorphan may have no therapeutic effects on cardiometabolic outcomes. In a meta-analysis of RCTs investigating the effects of Food and Drug Administration-approved weight-loss medications (i.e., orlistat, lorcaserin, naltrexone-bupropion, phentermine-topiramate, and liraglutide), Khera and colleagues (2018) found that these medications had only modest positive effects on cardiometabolic risk profile, e.g., FPG, cholesterol^34^. The weight-gain prevention effect of samidorphan may be too small to impact cardiometabolic parameters.

Caution should be applied in viewing the greater weight-gain prevention of samidorphan in individuals with lower initial BMI. Previous studies have shown inconsistent results regarding the association between the weight-loss efficacy of anti-obesity medications and initial weight or BMI. Liraglutide seems to be less effective in patients with high BMI (≥ 40 kg/m^2^)^35^. However, sibutramine-treated patients with higher initial weight could achieve greater weight loss than those with lower initial weight^36^. It remains unknown why the efficacy pattern of samidorphan is similar to that of liraglutide. This meta-regression finding should be, therefore, considered as a provisional result.

This meta-analysis found the negative results of olanzapine/samidorphan, but these results still help physicians and patients understand more about the risks and benefits of this medication. Although the pre-clinical data suggest that opioid antagonists can induce weight loss, the clinical data remain insufficient to support the use of olanzapine/samidorphan in clinical practice. More high-quality trials are warranted to support the benefits of samidorphan treatment for olanzapine-treated patients. Based on the findings of this study, future trials should focus on non-obese women with schizophrenia, who may respond to the weight-gain prevention effect of samidorphan more than obese men counterparts. In addition, weight and cardiometabolic outcomes should be assessed after several months of olanzapine/samidorphan treatment.

There were some limitations of this systematic review. First, the small number of included trials was the major drawback of this study. The statistical powers to detect the funnel plot asymmetry of publication bias and a significant correlation in meta-regression analysis are usually low when a meta-analysis involves fewer than ten studies^22,37^. Therefore, these few trials (n = 4) might contribute to the non-significant findings of publication bias and meta-regression analysis (e.g., no moderating effect of age or study duration on the treatment outcomes). Second, some participants’ characteristics might be the confounders in this study, e.g., two different groups of participants (healthy individuals vs. patients with schizophrenia), all healthy individuals being male. These confounders should also be taken into account in interpreting the meta-analytic and meta-regression results of this study. Third, the trial lengths of 3–24 weeks might be too short to determine the maximum efficacy of samidorphan in preventing weight gain. In addition, olanzapine-treated patients may gain weight continuously for up to 1–2 years^38^. Although we found a 36- to 60-week randomized trial in patients with schizophrenia comorbid alcohol use disorder, this trial compared only the changes of psychopathology and alcohol use between olanzapine/samidorphan and olanzapine/placebo groups^27^. Fourth, gender differences in obesity may limit the generalization of present findings to women with schizophrenia. While women with schizophrenia are more likely to have obesity than men^39^, only a small number of female patients were included in this study. While men appear to have a more favorable response to weight-loss interventions than women^40^, our meta-regression results suggest that olanzapine/samidorphan seems to express more benefit in preventing weight gain in women. Fifth, all trials included in this study were sponsored by a pharmaceutical company holding the patent of olanzapine/samidorphan, which might be associated with suspected reporting bias^41^. Last, the present findings should be generalized to patients with schizoaffective and bipolar disorder with caution. These patients may receive olanzapine treatment, but they have a higher risk of obesity than patients with schizophrenia^42^.

In conclusion, current clinical evidence may be insufficient to support the benefit of samidorphan in mitigating olanzapine-induced weight gain and metabolic syndrome. Samidorphan is well tolerated and well accepted by olanzapine-treated patients. More high-quality trials are warranted to support the benefits of samidorphan treatment for olanzapine-treated patients.

## Supplementary Information

Supplementary Information.

## Data Availability

The data and r script of this work are available at https://osf.io/g49sk/.
