# Supplementary material for: A meta-analysis comparing short-term weight and cardiometabolic changes between olanzapine/samidorphan and olanzapine
Source: Sci Rep. 2021 Apr 7;11:7583. doi: 10.1038/s41598-021-87285-w (PMC8027382; doi:10.1038/s41598-021-87285-w)
Supplement: Supplementary file 1 — Supplementary Information. [file 41598_2021_87285_MOESM1_ESM.pdf]

Supplementary Information

A Meta-Analysis Comparing Weight and Cardiometabolic Changes Between the Short-Term Treatments of Olanzapine/Samidorphan and Olanzapine

Manit Srisurapanont[1,2*], Sirijit Suttajit[1,2], Surinporn Likhitsathian[1], Benchalak Maneeton[1], Narong Maneeton[1]

[1] Department of Psychiatry, Faculty of Medicine, Chiang Mai University, Chiang Mai, Thailand

[2] These authors contributed equally: Manit Srisurapanont and Sirijit Suttajit

***Corresponding Author:**

Manit Srisurapanont (ORCID ID: 0000-0001-6203-1206)

Department of Psychiatry, Chiang Mai University Faculty of Medicine

110 Inthawarorot Road, Si Phum, Mueang, Chiang Mai 50200 THAILAND

Email: manit.s@cmu.ac.th

Appendix 1. Checklist of items to include when reporting a systematic review (with or w1ithout meta-analysis).

Section/Topic	#	Checklist Item	Reported on Page #
**TITLE**			
Title	1	Identify the report as a systematic review, meta-analysis, or both.	1
**ABSTRACT**			
Structured summary	2	Provide a structured summary including, as applicable: background; objectives; data sources; study eligibility criteria, participants, and interventions; study appraisal and synthesis methods; results; limitations; conclusions and implications of key findings; systematic review registration number.	2
**INTRODUCTION**			
Rationale	3	Describe the rationale for the review in the context of what is already known.	3-4
Objectives	4	Provide an explicit statement of questions being addressed with reference to participants, interventions, comparisons, outcomes, and study design (PICOS).	4
**METHODS**			
Protocol and registration	5	Indicate if a review protocol exists, if and where it can be accessed (e.g., Web address), and, if available, provide registration information including registration number.	4
Eligibility criteria	6	Specify study characteristics (e.g., PICOS, length of follow-up) and report characteristics (e.g., years considered, language, publication status) used as criteria for eligibility, giving rationale.	4-5
Information sources	7	Describe all information sources (e.g., databases with dates of coverage, contact with study authors to identify additional studies) in the search and date last searched.	5
Search	8	Present full electronic search strategy for at least one database, including any limits used, such that it could be repeated.	5
Study selection	9	State the process for selecting studies (i.e., screening, eligibility, included in systematic review, and, if applicable, included in the meta-analysis).	5
Data collection process	10	Describe method of data extraction from reports (e.g., piloted forms, independently, in duplicate) and any processes for obtaining and confirming data from investigators.	5
Data items	11	List and define all variables for which data were sought (e.g., PICOS, funding sources) and any assumptions and simplifications made.	5-6
Risk of bias in individual studies	12	Describe methods used for assessing risk of bias of individual studies (including specification of whether this was done at the study or outcome level), and how this information is to be used in any data synthesis.	6

Section/Topic	#	Checklist Item	Reported on Page #
Summary measures	13	State the principal summary measures (e.g., risk ratio, difference in means).	6
Synthesis of results	14	Describe the methods of handling data and combining results of studies, if done, including measures of consistency (e.g., I^2) for each meta-analysis.	6
Risk of bias across studies	15	Specify any assessment of risk of bias that may affect the cumulative evidence (e.g., publication bias, selective reporting within studies).	6
Additional analyses	16	Describe methods of additional analyses (e.g., sensitivity or subgroup analyses, meta-regression), if done, indicating which were pre-specified.	6-7
**RESULTS**			
Study selection	17	Give numbers of studies screened, assessed for eligibility, and included in the review, with reasons for exclusions at each stage, ideally with a flow diagram.	7
Study characteristics	18	For each study, present characteristics for which data were extracted (e.g., study size, PICOS, follow-up period) and provide the citations.	7
Risk of bias within studies	19	Present data on risk of bias of each study and, if available, any outcome-level assessment (see Item 12).	7
Results of individual studies	20	For all outcomes considered (benefits or harms), present, for each study: (a) simple summary data for each intervention group and (b) effect estimates and confidence intervals, ideally with a forest plot.	8
Synthesis of results	21	Present results of each meta-analysis done, including confidence intervals and measures of consistency.	8-9
Risk of bias across studies	22	Present results of any assessment of risk of bias across studies (see Item 15).	9
Additional analysis	23	Give results of additional analyses, if done (e.g., sensitivity or subgroup analyses, meta-regression).	9
**DISCUSSION**			
Summary of evidence	24	Summarize the main findings including the strength of evidence for each main outcome; consider their relevance to key groups (e.g., health care providers, users, and policy makers).	10
Limitations	25	Discuss limitations at study and outcome level (e.g., risk of bias), and at review level (e.g., incomplete retrieval of identified research, reporting bias).	12
Conclusions	26	Provide a general interpretation of the results in the context of other evidence, and implications for future research.	12
**FUNDING**			
Funding	27	Describe sources of funding for the systematic review and other support (e.g., supply of data); role of funders for the systematic review.	18

Appendix 2. Database searches on Oct 30, 2020.

Pubmed n=11
(samidorphan) AND (olanzapine) AND (weight OR obesity OR glucose OR sugar OR cholesterol, triglyceride) AND (random*)

Embase n=37
('samidorphan'/exp OR samidorphan) AND ('olanzapine'/exp OR olanzapine) AND ('weight'/exp OR weight OR 'obesity'/exp OR obesity OR 'glucose'/exp OR glucose OR 'sugar'/exp OR sugar OR 'cholesterol, triglyceride' OR (cholesterol, AND ('triglyceride'/exp OR triglyceride))) AND random*

Cochrane Library Trials n=31
(samidorphan) AND (olanzapine) AND (weight OR obesity OR glucose OR sugar OR cholesterol, triglyceride) AND (random*) in Title Abstract Keyword - (Word variations have been searched)

Additional searches at ClinicalTrial.gov and EudraCT n=4

Total N=83

Appendix 3. Subgroup meta-analysis (healthy participants and patients with schizophrenia) of FPG changes expressed as the standardized mean differences comparing between olanzapine/samidorphan and olanzapine.

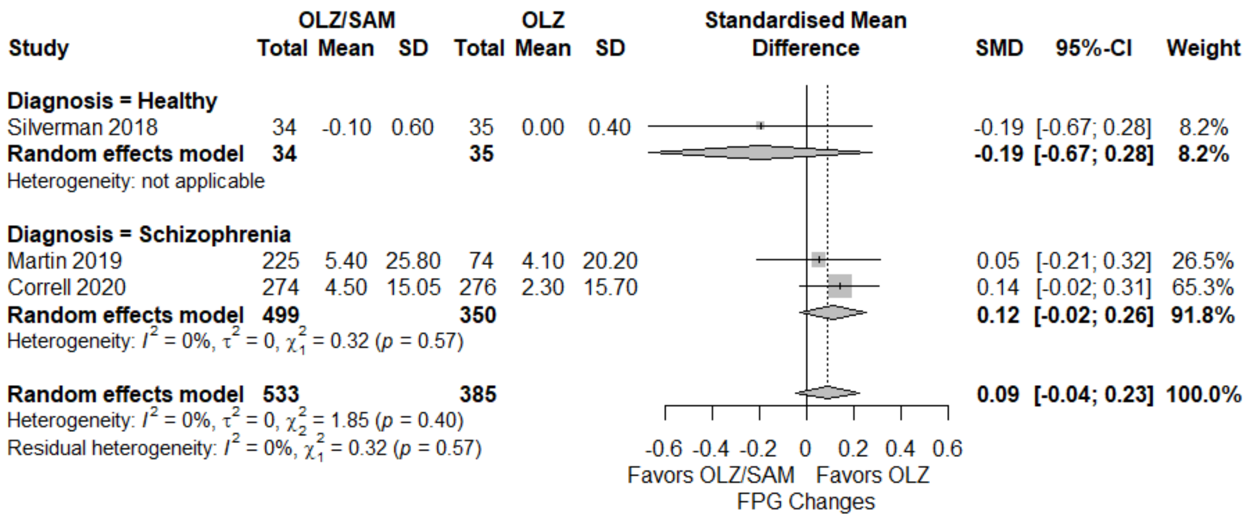

Appendix 4. Subgroup meta-analysis (healthy participants vs. patients with schizophrenia) of HDL changes expressed as the standardized mean differences comparing between olanzapine/samidorphan and olanzapine. The upper two diamonds indicate the pooled results of healthy and schizophrenia subgroups, respectively. The diamond at the bottom shows the pooled results of all trials.

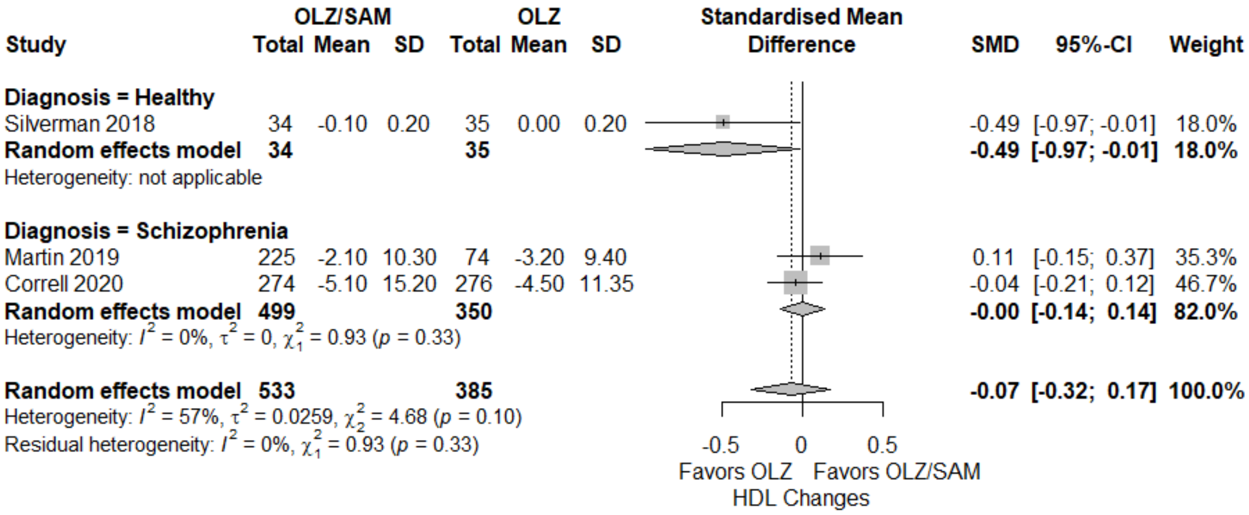

Appendix 5. Subgroup meta-analysis (healthy participants vs. patients with schizophrenia) of LDL changes expressed as the standardized mean differences comparing between olanzapine/samidorphan and olanzapine. The upper two diamonds indicate the pooled results of healthy and schizophrenia subgroups, respectively. The diamond at the bottom shows the pooled results of all trials.

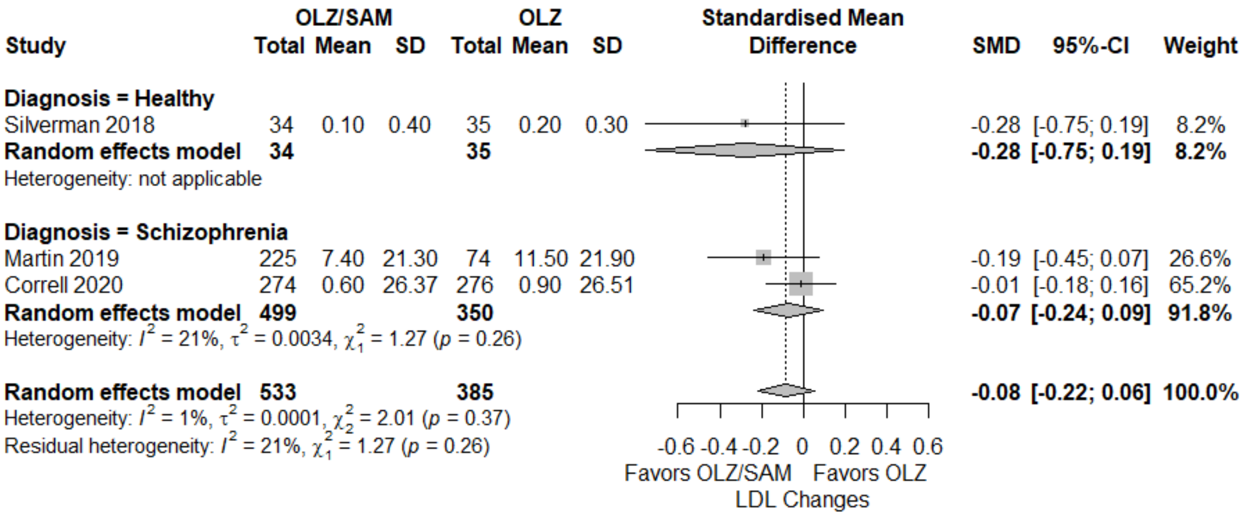

Appendix 6. Subgroup meta-analysis (healthy participants vs. patients with schizophrenia) of TG changes expressed as the standardized mean differences comparing between olanzapine/samidorphan and olanzapine. The upper two diamonds indicate the pooled results of healthy and schizophrenia subgroups, respectively. The diamond at the bottom shows the pooled results of all trials.

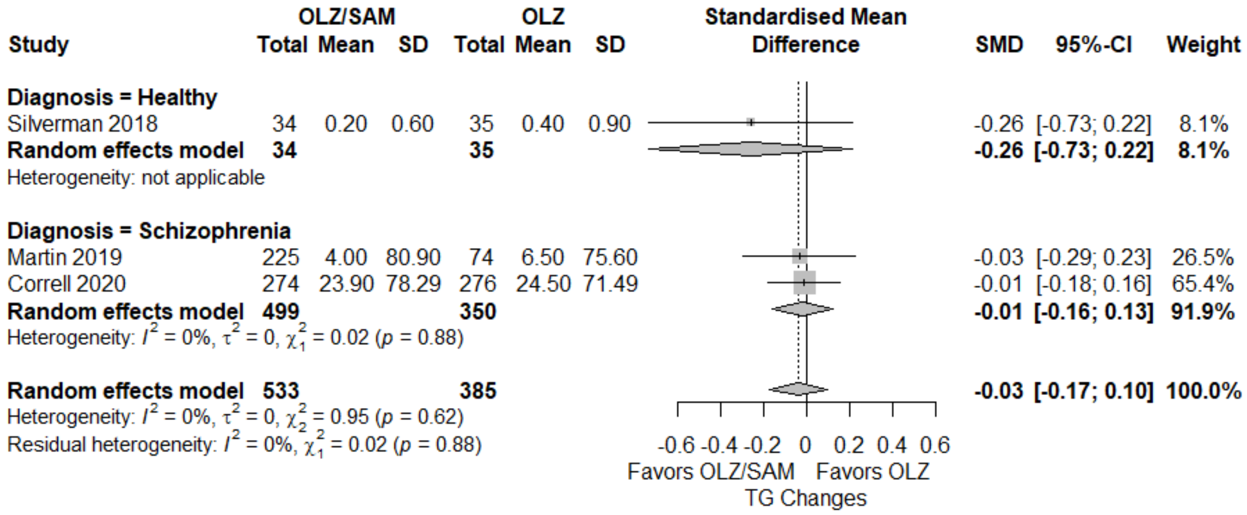

Appendix 7. Subgroup meta-analysis (healthy participants vs. patients with schizophrenia) of adverse dropout rates expressed as the risk ratios comparing between olanzapine/samidorphan and olanzapine. The upper two diamonds indicate the pooled results of healthy and schizophrenia subgroups, respectively. The diamond at the bottom shows the pooled results of all trials.

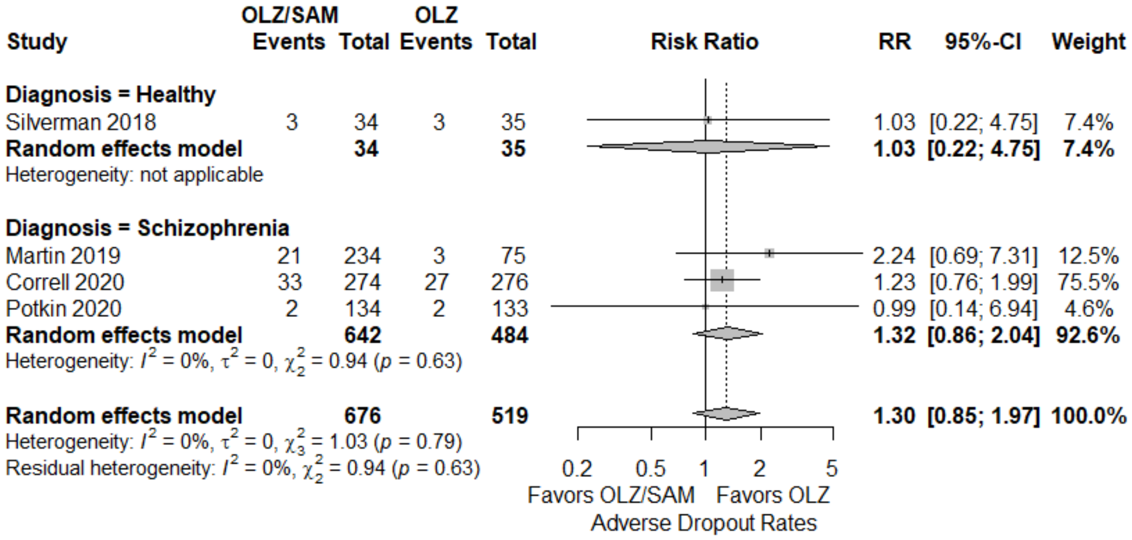

Appendix 8. Subgroup meta-analysis (healthy participants vs. patients with schizophrenia) of all-cause dropout rates expressed as the risk ratios comparing between olanzapine/samidorphan and olanzapine. The upper two diamonds indicate the pooled results of healthy and schizophrenia subgroups, respectively. The diamond at the bottom shows the pooled results of all trials.

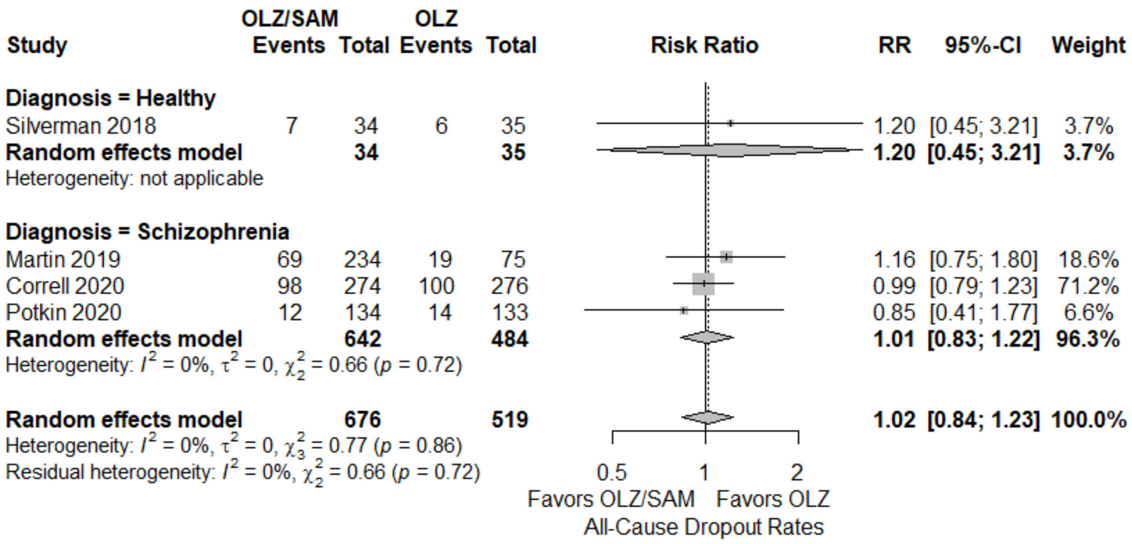

Appendix 9. Funnel plot for the publication bias of outcomes: (A) All-cause dropout rates, (B) FPS changes, (C) HDL changes, (D) LDL changes, (E) TG changes, and (F) Adverse dropout rates. Bold dash line indicates the random effects estimate.

(A) All-cause dropout rates

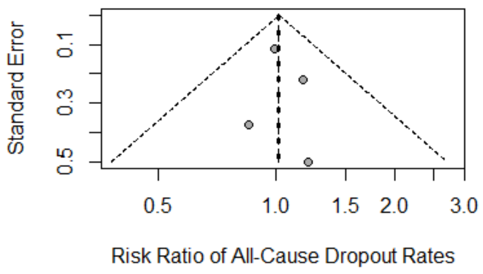

(B) FPS changes

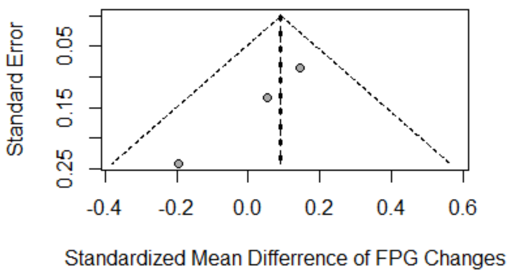

(C) HDL changes

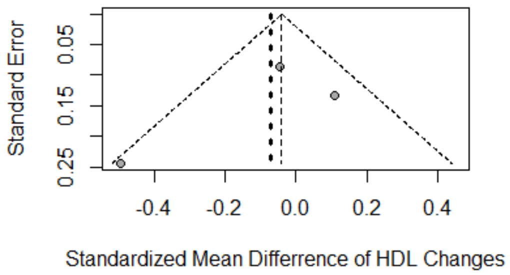

(D) LDL changes

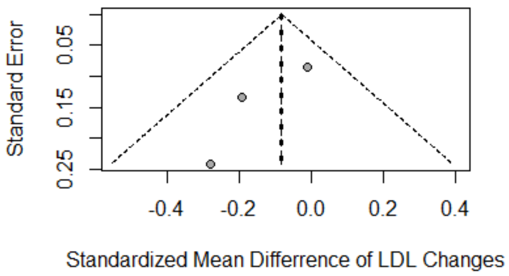

(E) TG changes

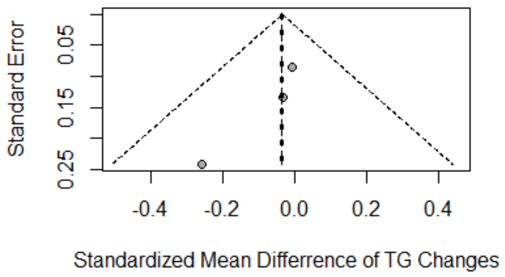

(F) Adverse dropout rates

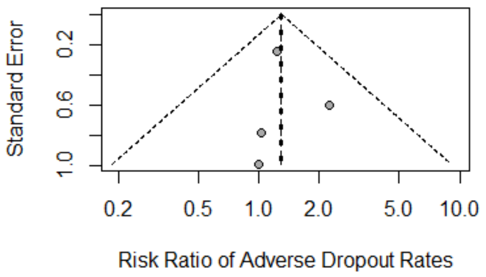

(G) All-cause dropout rates

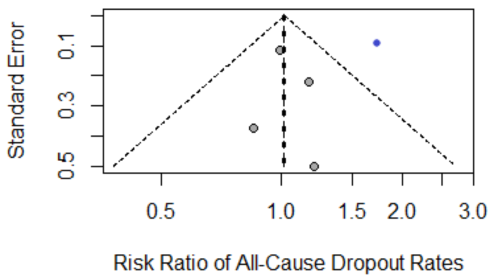

Appendix 10. GRADE approach used for rating the quality of cumulative evidence derived from the meta-analysis[a]

Dataset (study type= quality level)	Issues of concern that result in downgrading the evidence					Quality of evidence
	i) High risk of bias	ii) High inconsistency	iii) Indirectness	iv) Imprecision	v) High or indeterminable publication	
Weight change (RCTs=high)	No	Yes (-1 level)	No	Yes (-1 level)	Yes (-1 level)	Very low
FPG change (RCTs=high)	No	No	No	Yes (-1 level)	Yes (-1 level)	Low
HDL change (RCTs=high)	No	No	No	Yes (-1 level)	Yes (-1 level)	Low
LDL change (RCTs=high)	No	No	No	Yes (-1 level)	Yes (-1 level)	Low
TB change (RCTs=high)	No	No	No	Yes (-1 level)	Yes (-1 level)	Low
All-cause dropouts (RCTs=high)	No	No	No	Yes (-1 level)	Yes (-1 level)	Low
Adverse dropouts (RCTs=high)	No	No	No	Yes (-1 level)	Yes (-1 level)	Low

[a] Four levels of evidence quality include high, moderate, low, and very low.
RCT: randomized-controlled trial; FPG: Fasting plasma glucose; HDL: High-density cholesterol; LDL: Low-density cholesterol; TG: Triglyceride.

Adapted from Balshem, H. et al. GRADE guidelines: 3. Rating the quality of evidence. J. Clin. Epidemiol. 64, 401–406 (2011).
